# Concordance between self-reported body mass index with weight perception, self-rated health and appearance satisfaction in people living in Tehran

**DOI:** 10.1186/s40200-016-0244-8

**Published:** 2016-07-09

**Authors:** Arezoo Haghighian Roudsari, Abouali Vedadhir, Naser Kalantari, Parisa Amiri, Nasrin Omidvar, Hassan Eini-Zinab, Seyed Fatemeh Abdollah pouri Hosseini

**Affiliations:** 1 Nutritional Sciences, National Nutrition and Food Technology Research Institute, Faculty of Nutrition Sciences and Food Technology, Shahid Beheshti University of Medical Sciences, Tehran, Iran; 2 Department of Anthropology, Faculty of Social Sciences, Tehran University, Tehran, Iran; 3 Department of Community Nutrition, National Nutrition and Food Technology Research Institute, Faculty of Nutrition Sciences and Food Technology, Shahid Beheshti University of Medical Sciences, No. 7, Hafezi (West Arghavan) St., Farahzadi Blvd., Qods Town, 1981619573 P.O. Box 19395-4741, Tehran Iran; 4 Department of Social Determinants of Health, Research Institute of Endocrine Sciences, Shahid Beheshti University of Medical Sciences, Tehran, Iran

**Keywords:** Self-reported, Body mass index, Weight perception, Self-rated health, Appearance satisfaction

## Abstract

**Background:**

Obesity is investigated as a health concern due to high prevalence in the world. Nowadays, researchers are looking for an indirect method to measure weight and height. Self-reported Body Mass Index (BMI) is ever more served as an alternative method for direct weight and height measurement. Misreporting is a usual concern in self-reported BMI, thus, this study set explored the association and degree of agreement of self-reported BMI with weight perception, Self-Rated Health (SRH), and appearance satisfaction in people living in Tehran, Iran.

**Methods:**

722 men and women (268 men and 454 women) aged 30–64 years were selected using Cluster Multi-stage Sampling with the Probability Proportional to Size (PPS) method from each area. The questionnaire included demographic and socioeconomic variables and self-reported weight and height and questions related to weight and health perception, and appearance satisfaction. Independent samples *T*-test compared the mean of scales and differences in characteristics between BMI categories, analyzed using chi-square test. The *Cohen’s kappa* coefficient examined the association between self-reported BMI and weight perception, SRH, and appearance satisfaction.

**Results:**

The mean self-reported weight was 80.79 ± 12.87 in men and 68.33 ± 11.53 in women. The results of the agreement analysis for weight perception were Kappa = 0.38 with *p <* 0.0001 for women and Kappa = 0.23 with *p <*0.0001 for men. This measure of agreement, while statistically significant, is fair agreement. SRH and appearance satisfaction were not significantly correlated with self-reported BMI.

**Conclusion:**

The measurements of height and weight can cause significant imprecisions in calculation of BMI, which is used as a guide for identifying persons at risk of disease. Direct measurement of height and weight should be performed whenever possible for optimal measurements in clinical practice and clinically oriented researches.

## Background

Obesity is a growing health challenge in the world which consider as an important risk factor for many diseases like cardiovascular disease, diabetes and cancer [1]. There is high prevalence of overweight and obesity in all age groups in Eastern Mediterranean Region (EMR) [2], and obesity is more prevalent among women than men [3]. In Iran as a EMR countries, the prevalence of overweight and obesity 5.4 % and 1.6 %, respectively [4] and the mean BMI report as 24.9 and 26.5 kg/m^2^ in Iranian males and females, respectively [5]. Perceived body image, cultural meaning, and food subsidize plan, nutrition transition, inactivity, urbanization and an increase in the frequency of eating out, are main factors leading to obesity in this region [4].

Over the last 30 years, body image issues have increased and not only concern young people, but affect all people [6]. Body Image Dissatisfaction (BID) induced the adverse appraisals of body size and weight, in addition to dissimilarity between their perceived body shape and ideal body [7]. Overemphasis on fitness may lead to unhealthy dieting and eating disorders, whereas underestimation may increase the risk of the development of overweight and obesity [8]. The results of a study on body image demonstrated that modernity, awareness about globalization, life style, negotiation in family, cultural capital, and marriage status may account for about 40 % of Iranian women’s body image [9].

Researchers are looking for the valid indicators that could serve to predict the overweight and obesity prevalence. For adults, all studies have used Body Mass Index (BMI) recommended by World Health Organization (WHO) [2]. Validated self-reported BMI is ever more served as an alternative method for direct weight and height measurement which could spare the costs and time [10, 11]. Generally, under- and over-reporting is seen in self-reported weight and height, respectively. Obese women usually under-report their weight and over-report their height, so self-reported data may be inaccurate. It was observed more error in self-reported weight in overweight female than men and inaccurate reports in height are more likely in overweight status. Misreporting is a usual concern in self-reported BMI which could distort the research results and have negative effects on disease management and health status within the community [12].

This study set explores the association and degree of agreement of self-reported BMI with weight perception, Self-Rated Health (SRH) and appearance satisfaction in people living in Tehran, Iran.

## Methods

### Sample selection

This study is the second phase of a Mix Methods (MMs) research design, exploring the influencing factors of food choice in people living in Tehran, Iran. In the current quantitative phase, 722 men and women aged 30–64 years were selected using Cluster Multi-Stage Sampling via Probability Proportional to Size (PPS) method. Accordingly, for each selective area of Tehran, the sample size was determined proportional to its population. Tehran as the capital of Iran includes 22 districts with different socio-economic positions (SEP). For the purpose of the study, Tehran was divided into five geographic regions namely north, south, west, east, and center. For every geographic region, two districts were selected (10 districts out of 22) randomly.

The questionnaire included issues on demographic and socioeconomic variables and self-reported weight and height. Other questions related to weight and health perception and appearance satisfaction were developed by a group of experts in nutrition, statistics, health education, and sociology. The socioeconomic variables were age, gender, occupation status, education, and duration of residence in Tehran, ethnicity, religion, marital status, and the number of children.

### BMI classification

The Quetelet index or BMI was used to evaluate the nutritional status of subjects, calculated by the ratio between weight (kg) and height (m^2^), which is better correlated to the weight and fatty mass [13, 14], and it is the most valid criterion to estimate the increase in weight, adiposity and obesity [15]. This index categorized as underweight (BMI < 20), normal weight (BMI 20 - <24.9), overweight (BMI 25 – <30), and obesity (BMI ≥ 30) [2]. In our study, we calculated the BMI by self-reported weight and height.

### Weight perception

Subjects were asked to report their views on body weight on five statuses: “thin”, “underweight”, “normal weight”, “overweight”, and “obese”. The body-weight perception of the participants was assessed asking the following question: *“How would you describe your current weight?”*

### Self-rated health (SRH)

SRH was obtained through this question: *“Totally, would you say your health is laid in which status: very healthy, healthy, weakling, sick (unhealthy)?”* Subjects’ susceptibility of diseases was asked using Multiple Response Set (MRS) of diseases such as cardiovascular diseases, hypertension, diabetes, osteoporosis, hyperlipidemia, cancer, and arthritis, eye, gastrointestinal, oral, endocrine, and mental diseases to evaluate the real health status. According to definition of chronic diseases, we categorized cardiovascular disease, diabetes, arthritis and cancer as “chronic diseases” [16].

### Appearance satisfaction

The following question was asked to evaluate the rate of appearance satisfaction: *“How satisfied are you from your appearance?”* The participants stated their satisfaction on these levels: *“very much, much, moderate, little, very little”*.

### Statistical analysis

The IBM SPSS software (version 21) was used for statistical analysis of the data. Independent samples *T*-test compared the mean of scales. Differences between BMI categories were analyzed using Chi-square test. A reliability using the Cohen’s Kappa statistic was performed to determine consistency among self-reported BMI and weight perception, SRH, and appearance satisfaction. In all analyses, *P*-values < 0.05 were considered statistically significant.

## Results

### Socio-demographic characteristics of respondents

The general characteristics of respondents are summarized in Table 1. Of 722 participants, 268 were men and 454 were women with the mean age of 42.19 ± 9.48 (yrs.). Totally, the mean self-reported weight was 72.97 ± 13.46 kg; the mean self-reported height was 166.80 ± 9.72 cm and the mean self-reported BMI was 26.22 ± 4.22 kg/m^2^. In terms of marital status, 79.4 % of subjects were married, 26.9 % of them were single and 4 % of subjects was divorced or widowed which about 73 % of participants had children.

**Table 1:** Socio-demographic characteristics of respondents

Demographic and socioeconomic	Men (*n =* 268)	Women (*n =* 454)		Total (valid)	
Variables	Mean ± SD	Mean ± SD			
*Age* (yrs.)	43.1 ± 9.8	41.6 ± 9.1		722 (100)	
*Self-reported Weight*	80.79 ± 12.87	68.33 ± 11.53		720 (99.7)	
*Self-reported Height*	175.15 ± 7.5	161.83 ± 7.21		712 (98.6)	
*Monthly cost (USD)*	719 ± 389	613 ± 396		659 (91.2)	
*Income (USD)*	931 ± 550	730 ± 533		625 (86.5)	
*Duration of residency in Tehran*	31.6 ± 14.6	32.3 ± 14.3		703 (97.3)	
	Men (*n =* 268)	Women (*n =* 454)	Total (valid)	*X* ^*2*^ *value*	p*
	n (%)	n (%)			
*Education*					
Illiterate	0 (0)	4 (0.9)			
Primary school	13 (4.9)	16 (3.5)	717	19.11	
High school	71 (26.7)	171 (37.9)			<0.001*
Undergraduate	117 (44)	196 (43.5)			
Graduate	65 (24.4)	64 (14.2)			
*Occupation Status*					
Unemployed	6 (2.2)	11 (2.4)			
Employed	141 (52.6)	170 (37.5)			
Housekeeper	0 (0)	212 (46.8)	721	217.93	
Retired	41 (15.3)	32 (7.1)			< 0.0001*
University student	7 (2.6)	10 (2.2)			
Self-employed	73 (27.2)	18 (4)			
*Marital status*					
Single	50 (18.7)	70 (15.4)			
Married	213 (79.5)	360 (79.3)	722	8.97	< 0.03*
Divorced	5 (1.9)	11 (2.4)			
Widowed	0 (0)	13 (2.9)			
*Children*					
Yes	186 (35.4)	339 (64.6)	718	2.58	0.1
No	81 (42)	112 (58)			
*History of Chronic diseases*					
CVD	19 (7.1)	20 (4.4)		2.37	0.08
Diabetes	11 (4.1)	32 (7)		2.60	0.07
Arthritis	13 (4.9)	61 (13.4)	722	13.5	< 0.0001*
Cancer	0 (0)	2 (0.4)		1.18	0.30
*History of Chronic disease risk factors*					
Hypertension	30 (11.2)	38 (8.4)		1.57	0.13
High Blood Cholesterol	33 (12.3)	49 (10.8)		0.38	0.30

*p < 0.05 is significant

### Self-reported BMI and weight perception

The mean self-reported BMI of each age group is presented in Table 2. The total percent of overweight and obesity prevalence was 60.5 and 54.9 in men and women. The high percentage of men in all age groups was overweight, versus half of the women aged 41–50 years old were in normal BMI group. The most obese age group was women in their thirties, and the most overweight age group was women aged 51–64 years old. Normal weight women described themselves as either too thin or underweight or normal weight and more overweight men perceived their weight via self-reported BMI as overweight. This was also observed in overweight women, so that more than half of these women described themselves as overweight (Table 4). However, 77.8 % of women who perceived themselves as thin or underweight had the normal self-reported BMI.

**Table 2:** Self-reported BMI by age and sex

Variables	*Body Mass Index calculated by self-reported weight and height (kg/m2)*				*X* ^*2*^ *value*	*p* ^*a*^
	Underweight BMI < 20	Normal 20 < BMI < 24.9	Overweight 25 < BMI <29.9	Obesity BMI ≥ 30		
*Male*						
30–40 yr old (*n* = 135)	3 (2.2)	50 (37)	59 (43.7)	23 (17)		
41–50 yr old (*n* = 65)	0 (0)	25 (38.5)	29 (44.6)	11 (16.9)	5.59	0.69
51–64 yr old (*n* = 61)	0 (0)	21 (31.8)	32 (52.5)	8 (13.1)		
Total (*n* = 261)						
*Women*						
30–40 yr old (*n* = 109)	7 (2.9)	31 (28.4)	44 (40.4)	33 (30.3)		
41–50 yr old (*n* = 239)	1 (0.9)	120 (50.2)	79 (33.1)	33 (13.8)	38.23	< 0.0001*
51–64 yr old (*n* = 88)	0 (0)	28 (31.8)	49 (55.7)	11 (12.5)		
Total (*n* = 436)						

^*a*^Chi-Square

*p < 0.05 is significant

### Chronic diseases and SRH

We categorized the subjects into 4 groups based on the number of chronic diseases which they suffered from them. These groups include no diseases, one or two or three prominent chronic diseases according to self-reporting. The incidence of chronic diseases was 5.4, 6, 10.2 and 0.3 % for CVD, diabetes, arthritis and cancer, respectively. In terms of given disease, the percentage of women who reported one or two or three chronic diseases, was more than men so that 79.3 % of women had no chronic disease, whereas this rate was 85.1 % for men. Despite of this self-report of diseases, SRH showed significant difference regarding age groups which this difference was higher in women compared to men (*p <* 0.0001 vs. < 0.02); namely, women perceiving themselves as healthy and very healthy in their thirties (Table 3). Moreover, to explore the possible impact of diseases’ history on the relationship of self-reported BMI and SRH, the chi-square test was repeated in the subjects with or without chronic diseases by sex groups. There was no difference between two analyses in both men and women.

**Table 3:** Weight perception, SRH and appearance satisfaction of respondent by age and sex

variables	*Age group of men (Years)*			*X* ^*2*^ *value* ^*a*^	p*	*Age group of women (Years)*			*X* ^*2*^ *value* ^*a*^	p*
	30–40	41–50	51–64			30–40	41–50	51–64		
*Weight perception*										
Thin Underweight Normal weight Overweight Obese	3 (50) 10 (58.8) 74 (51.4) 47 (48.5) 4 (100)	2 (33.3) 2 (11.8) 33 (22.9) 29 (29.9) 0 (0)	1 (16.7) 5 (29.4) 37 (25.7) 21 (21.6) 0 (0)	7.46	0.4	8 (88.9) 6 (66.7) 104 (57.8) 115 (50.9) 16 (55.2)	0 (0) 2 (22.2) 37 (20.6) 62 (27.4) 10 (34.5)	1 (11.1) 1 (11.1) 39 (21.7) 49 (21.7) 3 (10.3)	10.89	0.2
*Self-rated Health*										
Sick (Unhealthy) Weakling Healthy Very Healthy	2 (28.6) 5 (29.4) 90 (48.6) 41 (69.5)	2 (28.6) 5 (29.4) 48 (25.9) 11 (18.6)	3 (42.9) 7 (41.2) 47 (25.4) 7 (11.9)	14.44	< 0.02*	2 (7.4) 40 (53.3) 154 (56.2) 54 (71.1)	11 (40.7) 20 (26.7) 64 (23.4) 15 (19.7)	14 (51.9) 15 (20) 56 (20.4) 7 (9.2)	36.42	< 0.0001*
*Appearance satisfaction*										
Very Little Little Moderate Much Very Much	2 (40) 1 (20) 57 (47.1) 54 (54.5) 24 (63.2)	1 (20) 3 (60) 29 (24) 26 (26.3) 7 (18.4)	2 (40) 1 (20) 35 (28.9) 19 (19.2) 7 (18.4)	9.28	0.3	5 (45.5) 9 (50) 120 (50.4) 83 (62.9) 32 (62.7)	3 (27.3) 7 (38.9) 60 (25.2) 28 (21.2) 11 (21.6)	3 (27.3) 2 (11.1) 58 (24.4) 21 (15.9) 8 (15.7)	10.27	0.2

^*a*^Chi-Square

*p < 0.05 is significant

Based on the self-reported BMI, the men who rated themselves as sick (unhealthy) were overweight and obese. According to Table 4, the majority of overweight men described their health status as weakling, healthy and very healthy; however, about 50 % of women who perceived themselves as weakling had normal BMI. Very healthy, as SRH, was expressed less by overweight men compared to normal weight women perceiving themselves as very healthy.

**Table 4:** The rate of agreement between self-reported BMI and weight perception, SRH and appearance satisfaction by sex

Variables	*Body Mass Index calculated by self-reported weight* *(kg/m2) (Men)*				*Total*	*X* ^*2*^ *value* ^*a*^	*Body Mass Index calculated by self-reported weight* *(kg/m2) (Women)*				*Total*	*X* ^*2*^ *value* ^*a*^
	Underweight BMI < 20	Normal 20 < BMI < 24.9	Overweight 25 < BMI <29.9	Obesity BMI ≥ 30			Underweight BMI < 20	Normal 20 < BMI < 24.9	Overweight 25 < BMI <29.9	Obesity BMI ≥ 30		
*Weight perception*												
Thin-Underweight Normal weight Overweight Obese	2 (8.7) 1 (0.7) 0 (0) 0 (0)	19 (82.6) 74 (51.4) 3 (3.1) 0 (0)	2 (8.7) 59 (41.0) 58 (59.8) 1 (25)	0 (0) 6 (4.2) 33 (34.0) 3 (75)	23 140 94 4	126.17	4 (22.2) 4 (2.2) 0 (0) 0 (0)	14 (77.8) 123 (68.3) 41 (18.1) 1 (3.4)	0 (0) 39 (21.7) 126 (55.8) 6 (20.7)	0 (0) 6 (3.3) 49 (21.7) 22 (75.9)	18 172 216 29	257.22
*Cohen’s Kappa Coefficient*	0.23*					*p <* 0.0001**	0.38*					*p <* 0.0001**
*Self-rated Health*												
sick (unhealthy) weakling Healthy Very healthy	0 (0) 0 (0) 3 (1.7) 0 (0)	0 (0) 7 (41.2) 70 (39.1) 19 (32.2)	4 (66.7) 8 (47.1) 79 (44.1) 29 (49.2)	2 (33.3) 2 (11.8) 27 (15.1) 11 (18.6)	6 17 179 59	12.4	1 (3.8) 2 (2.7) 4 (1.5) 1 (1.4)	7 (26.9) 32 (43.8) 100 (38.2) 39 (53.4)	9 (34.6) 25 (34.2) 116 (44.3) 22 (30.1)	9 (34.6) 14 (19.1) 42 (16) 11 (15.1)	17 73 262 73	15.07
*Cohen’s Kappa Coefficient*	–0.007					*P =* 0.8	0.04					*P =* 0.1
*Appearance satisfaction*												
Very little-Little Moderate Much Very much	0 (0) 2 (1.7) 0 (0) 1 (2.6)	4 (40) 44 (36.4) 36 (36.4) 12 (31.6)	0 (0) 51 (42.1) 49 (49.5) 20 (52.6)	4 (40) 21 (17.4) 13 (13.1) 4 (10.5)	8 118 98 37	25.84	1 (3.4) 4 (1.7) 2 (1.5) 1 (2)	8 (27.6) 83 (34.9) 60 (45.5) 26 (51.0)	6 (20.7) 102 (42.9) 49 (37.1) 13 (25.5)	14 (48.3) 38 (16.0) 16 (12.1) 9 (17.6)	29 227 127 49	33
*Cohen’s Kappa Coefficient*	0.01					*P =* 0.6	–0.03					*P =* 0.2

^*a*^Chi Square

* Fair agreement

** *p* < 0.05 is significant

### Appearance satisfaction

Regarding to appearance satisfaction, the majority of men and women in all age group satisfied from their appearance moderately. Overweight men were more satisfied with their appearance as moderate, much, and very much, and normal weight women were satisfied with their appearance as much and very much. Table 4 shows that the obese women’ appearance satisfaction was very little or little.

### Degree of concordance

The kappa coefficient was significant for weight perception, and the amount of agreement between self-reported BMI and perceived weight was higher in women compared with men. The results of the agreement analysis were Kappa = 0.38 with *p <* 0.0001 for women and Kappa = 0.23 with *p <*0.0001 for men (Table 4). This measure of agreement, while statistically significant, is fair agreement. As a rule, Kappa values from 0.0 to 0.20 are considered slight agreement, 0.21 to 0.40 fair, and o.41 to 0.60 moderate. Cohen’s Kappa varies from 0 to 1.0 (although negative numbers are possible). When the value of kappa coefficient is closer to one, there is more agreement between the assessed variables. However, the values near or less than zero, suggest that agreement is attributable to chance alone [17]. Kappa coefficient showed no significant agreement between SRH and appearance satisfaction regarding self-reported BMI by sex.

### Subjects’ response rate

The response rate of subjects was 99.7 % for self-reported weight; 100 % of men and 99.5 % of women responded. The men answered the question on self-reported height slightly more than women (98.8 % vs. 98.4 %). The women were less likely to respond the questions about monthly costs (88.7 %) and income (83.7 %) than men (95.5 % and 91.4 %, respectively). All of men answered the questions about their perceived weight, health, and appearance. In contrast, of 454 women subjects, 453 (99.8 %) answered the question about their weight perception; 452 (99.6 %) reported their SRH, and 450 (99.1 %) described their satisfaction about appearance.

## Discussion

Self-reported BMI showed the high prevalence of overweight in men and normal weight in women compared with other BMI categories. In contradiction with previous studies which indicated the mean BMI 24.9 kg/m^2^ (normal) and 26.5 kg/m^2^ (overweight) in Iranian males and females, respectively [4], the current results showing the higher prevalence of overweight in men than women based on the self-reported BMI. These findings proposed that self-reported BMI may have some error in identifying the real BMI. A systematic review revealed trends of under-reporting for weight and BMI and over-reporting for height; although the degree of the trend was different for men and women [18]. Height overestimation and weight underestimation reported in women and great differences were observed between self-reporting and measuring the height and weight of several women based on the review article [19]. These findings explain the cause of the contradiction of the present results which showed that the men were more overweight than women, in spite of prevalence of overweight in Iran. The relationship between perceived weight status and self-reported BMI was moderate in another cross-sectional study [20].

The concordance between self-reported BMI and weight perception was significantly in both men and women found in our study. Weight perception in women is in line with self-reported BMI as normal, overweight, and obese category. Weight perception in overweight and obese men was according to self-reported BMI, and the women perceived their weight less than that their weight showed. Our data showed the women who had normal self-reported BMI perceived them as thin or underweight. In contrast with Japanese workers [21], the current results indicated a difference regarding the relationship between BMI categories and weight perception in men and women, i.e. based on kappa coefficient; the rate of agreement in women was greater than men (0.38 vs. 0.23). This finding is confirmed by higher percent of normal self-reported BMI in women than men, which could result in better weight perception in women. The association between BMI and weight perception of Japanese differed by age and gender [21]. A cross-sectional analysis among employees showed that the women considered themselves more to be overweight through each BMI category when compared with men [22]. Within weight perception categories, high percentage of women correctly perceived their weight as normal, overweight and obese in high degree of agreement with self-reported BMI.

It was found that the perception regarding weight, health, and appearance satisfaction varied according to gender and subjects’ age group. There were no significant differences among age groups within levels of weight perception and appearance satisfaction.

**Fig. 1:**
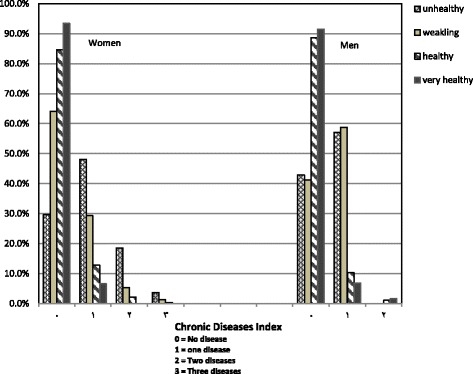
Comparison between percentage of men and women in terms of association of chronic diseases and self-rated health

Within age groups, only women in their thirties significantly perceived themselves healthy or very healthy (*p <* 0.0001) which probably due to absence of or little chronic diseases in this age decade. The results also revealed that the overweight men considered themselves healthy and very healthy, whereas normal weight women stated SRH as very healthy more. The correlation between SRH and self-reported BMI was examined through the agreement coefficient. Despite of the upper kappa coefficient in women compared to men, our data showed there was no significant agreement between SRH and self-reported BMI in each sex groups, which is contrast with the study of Giron’s on Spanish women who report SRH as good less than men [23]. SRH may be affected by disease status; based on self-report diseases, they suffered from a variety of disease and disabilities by gender. Thus, we explored the impact of chronic diseases’ history, as a possible confounder, on the association of self-reported BMI and SRH. This association was similar between two groups with and without chronic diseases by sex. Figure 1 showed the status of SRH in men and women who suffered from chronic disease.

Using SRH provides a valid, reliable, and cost-effective health assessment; particularly, in studies lacking other forms of health information [24, 25]. In our study, the pattern of SRH was similar to weight perception, so that the observed agreement between weight perceptions with self-reported BMI in women was also seen for SRH concordance with self-reported BMI, however, this agreement was not significant. Nevertheless, the results of a research on Swedish people demonstrated that the reliability of SRH is as good as or even better than more specific questions [26].

Our data revealed more overweight men were satisfied with their appearance, and women classified themselves better than men similar to European Union [11]. The majority of overweight men were meaningfully satisfied with their appearance as much or very much. According to the findings, the discordance between the men’s appearance satisfaction and self-reported BMI was more compared to women; however, the agreement coefficient was not significant in both gender groups. As Korean people who were not satisfied with their weight in spite of the fact that they had normal or lean figures, except for 3.3 % of the total samples [27]. Body image would be valid between 30 % of men and 60 % of women approximately; however, it had high validity in underweight males and females, [11]. The rate of appearance satisfaction in women was reduced with increasing the age. Body image is the important factor to evaluate women in societies like Iran and for this reason they are pushed to pay more attention to body shape and its management. Thus, their appearance satisfaction will be lower than men [9].

This study has some limitations which the most important being the lack of direct weight and height measurement. Because of extensive data collected in the main great design and a large number of subjects, this was not possible. These data could help the researchers to compare the self-reported and real measurements to conclude the better association between variables.

## Conclusion

These findings proposed the current measurements of height and weight can cause significant imprecision in calculation of BMI and used as a guide for identifying persons at risk of disease. On the other hand, direct measurement of height and weight is recommended to be performed whenever possible for optimal measurements in clinical practice and research [19]. Therefore, plan of actions should be prepared to promote health status with respect to accurate control of body weight in terms of different patterns by gender.

## Abbreviations

BMI, body mass index; SRH, self rated health; PPS, probability proportional to size; WHO, world health organization; EMR, Eastern Mediterranean Region; BID, body image dissatisfaction; MMs, mix methods; SEP, socio-economic position; MRS, multiple response set
